# Regression of Chiari malformation type 2 following early postnatal meningomyelocele repair—a retrospective observation from an institutional series of patients

**DOI:** 10.1007/s00381-024-06586-3

**Published:** 2024-08-29

**Authors:** Radek Frič, Mona Kristiansen Beyer, Bernt Johan Due-Tønnessen

**Affiliations:** 1 Dept. of Neurosurgery, Oslo University Hospital - Rikshospitalet, Sognsvannsveien 20, N-0027 Oslo, Norway; 2 Dept. of Radiology, Oslo University Hospital - Rikshospitalet, Oslo, Norway; 3 Faculty of Medicine, University of Oslo, Oslo, Norway

**Keywords:** Meningomyelocele, Arnold Chiari malformation, Spinal dysraphism

## Abstract

**Purpose:**

Spontaneous regression of Chiari malformation type 2 (CM2) is observed rarely, as CM2 is associated with meningomyelocele (MMC) that is surgically repaired either pre- or early postnatally. While the radiological regression of CM2 occurs frequently following prenatal repair of MMC, it has been reported in only a few studies after postnatal repair.

**Methods:**

From the consecutive series of children with postnatally repaired MMC, we reviewed the clinical and radiological data regarding CM2, particularly its regression either spontaneously or following CSF diversion.

**Results:**

Eighteen children underwent postnatal repair of MMC between February 2011 and April 2023. CM2 was present in 16 (89%), and hydrocephalus in 15 children (83%), requiring shunting in 14 of them. During the mean clinical observation time (from birth to April 2023) of 59 ± 51 months, three children with CM2 (19%) underwent 1–2 foramen magnum decompressions (FMD), five children (28%) 1–4 surgical untethering procedures and 13 children with shunted hydrocephalus (93%) 1–5 shunt revisions. Out of sixteen children with CM2, we observed regression of CM2 on MRI in only one case (6%) during the mean radiological follow-up (from birth to the last MRI taken) of 49 ± 51 months.

**Conclusion:**

In our experience, spontaneous regression of CM2 in children with postnatally repaired MMC occurs quite rarely. Pathophysiological mechanisms behind the development of CM2 in children with MMC remain unclear, but our observation supports the hypothesis of an association between the downward displacement of the hindbrain and the low intraspinal pressure secondary to CSF leakage in children born with MMC.

## Introduction

Following the original description of hindbrain herniation in the 1890s [1], referred to as Chiari malformation, several different types have later been recognized. In contrast to Chiari malformation type 1 (CM1), types 2–4 are typically associated with craniospinal dysraphism. Specifically, type 2 (CM2) is exclusively associated with an open form of spinal dysraphism, i.e. the meningomyelocele (MMC), where it occurs in 80–99% of cases [2–4]. From a pathoanatomical point of view, CM2 is characterized by a small posterior fossa and hindbrain herniation through the foramen magnum and into the cervical spinal canal, elongation and kinking of the medulla oblongata, resulting in the compression of the brainstem and obliteration of the fourth ventricle and cisterna magna.

Although requiring treatment in only a minority of cases, CM2 is the leading cause of mortality in the earliest period of life of children born with MMC [4–6].

The aetiology, pathophysiology and natural history of Chiari malformations are still poorly explored. One interesting observation in CM1 is that the cerebellar tonsillar ectopy into the foramen magnum—a typical radiological feature of CM1—may in children regress spontaneously, sometimes even completely. This phenomenon, yet still not very well explained, has been reported repeatedly and may occur in as much as 12% of children with CM1 [7].

In contrast, spontaneous regression of hindbrain herniation in CM2 is difficult to observe in real life, as it is always associated with meningomyelocele (MMC) that is surgically repaired either pre- or early postnatally. Furthermore, the majority of children require early diversion of cerebrospinal fluid (CSF).

While the radiological regression of CM2 seems to occur more frequently following prenatal repair of MMC [8–14], it has been observed in only a few studies after postnatal repair of MMC [3, 15–17], where some authors report a regression rate up to 40% [3]. In this report, we wished to identify similar observations in our series of children with postnatally repaired MMC.

## Methods

The study was approved by Oslo University Hospital as a quality control study (reference number 23/16883), waiving the need for informed consent.

From the institutional series of children with the postnatal repair of MMC treated with primary repair at Oslo University Hospital-Rikshospitalet in the period from 2011 to 2023, we reviewed the clinical and radiological data regarding associated CM2, particularly its radiological dynamics either spontaneously or following CSF diversion. A board-certified radiologist (M.K.B.) reviewed all available imaging.

## Results

During the study period, eighteen children (male/female ratio 1:1) were born with MMC and operated with the primary MMC repair during the first days (median 0 days, range 0–7) postnatally. The level of dysraphism was as follows: cervical (myelocystocele, *n* = 1), thoracal (*n* = 3), lumbal (*n* = 9), lumbosacral (*n* = 2) and sacral (*n* = 3), respectively (Table 1).

**Table 1:** Clinical and radiological details from the patient cohort in the present study (*n* = 18)

Patient	Sex	MMC level	CM2	Caudal position of vermis	Syrinx	HC	Shunt	CM2 regression	FMD	Shunt revision	Untethering	MRI FU (Months)	Observation (Months)
1	M	Th9-10	Yes	C4	Yes	Yes	Yes	No	No	No	1 ×	155	158
2	M	S2	No	-	No	No	No	-	-	-	3 ×	129	145
3	M	L3-4	Yes	C2	Yes	Yes	Yes	No	No	5 ×	4 ×	133	140
4	F	L3-4	Yes	C0	Yes	Yes	Yes	No	1 ×	1 ×	2 ×	98	106
5	F	Th8	Yes	C4	No	Yes	Yes	No	No	3 ×	No	95	100
6	F	S2	Yes	C2	Yes	Yes	Yes	No	No	1 ×	No	4	7
7	F	C5-6	Yes	C5	Yes	Yes	No	No	No	-	No	7	82
8	F	L3-4	Yes	C6	Yes	Yes	Yes	No	2 ×	2 ×	1 ×	69	75
9	M	L4-5	Yes	C3	No	Yes	Yes	No	No	3 ×	No	44	51
10	F	L5-S1	Yes	C4	No	Yes	Yes	No	No	1 ×	No	25	40
11	F	L5-S1	Yes	C1	No	No	No	**Yes**	No	-	No	24	36
12	F	Th12-L1	Yes	C4	Yes	Yes	Yes	No	No	1 ×	No	27	28
13	M	L4-5	Yes	C5	No	Yes	Yes	No	2 ×	3 ×	No	18	23
14	F	S3-4	No	-	No	No	No	-	-	-	No	14	20
15	M	L1	Yes	C3	Yes	Yes	Yes	No	No	1 ×	No	13	18
16	M	L2-3	Yes	C4	No	Yes	Yes	No	No	1 ×	No	8	15
17	M	L4-5	Yes	C5	No	Yes	Yes	No	No	2 ×	No	7	12
18	M	L3	Yes	C3	No	Yes	Yes	No	No	1 ×	No	12	12

*C*, cervical; *CM2*, Chiari malformation type 2; *FMD*, foramen magnum decompression; *FU*, follow-up; *HC*, hydrocephalus, *L*, lumbal, *LS*, lumbosacral; *MRI*, magnetic resonance imaging; *S*, sacral; *Th*, thoracal

Initial MRI was performed during the early postnatal period in all cases (between 0 and 10 days after birth, median 2 days), out of which before the surgical repair of MMC in six cases (33%). CM2 was verified by MRI in 16 (89%), and hydrocephalus in 15 children (83%), requiring treatment with a shunt in 14 of them. Syringomyelia was observed in 8 (44%) children.

One patient was lost to follow-up after 7 months, as the family moved abroad. During the mean clinical observation time (from birth to April 2023) of 59 ± 51 months, three children with CM2 (19%) underwent 1–2 foramen magnum decompressions (FMD) for CM2, five children (28%) 1–4 surgical untethering procedures and 13 children with shunted hydrocephalus (93%) 1–5 shunt revisions. In addition, one child underwent laminotomy Th 3–6 and fenestration of syrinx (patient no. 13 in Table 1). Another child underwent a minimally invasive suturectomy due to unilateral coronary craniosynostosis at the age of 4 months (patient no. 16 in Table 1).

Out of sixteen children with CM2, we could observe regression of CM2 features on MRI during the mean radiological follow-up (from birth to the last MRI taken) of 49 ± 51 months in only one single case (6%):

## Case report

The girl (patient no. 11 in Table 1) was born by spontaneous vaginal delivery in gestational week 39, with an Apgar score of 8–9-9, body weight of 3910 g, length of 49 cm and head circumference of 33 cm (p3-percentile). Flexion contractures in the knees and ankles were observed, with significantly reduced motoric function (flexion) in the lower extremities.

**Fig. 1:**
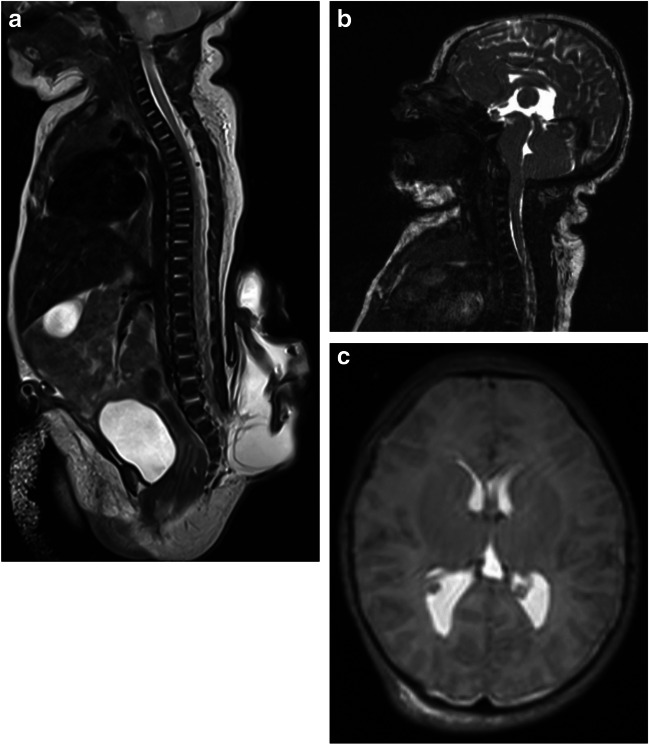
The initial MRI taken before the primary surgical repair of MMC, revealing **A** a lumbosacral MMC and **B** intracranially a mild variant of CM2 with reduced volume of posterior cranial fossa, beaked tectum, downward herniation of the vermis, and reduced cerebrospinal fluid (CSF) space in the foramen magnum, **C** while supratentorial ventricles were small

Due to the clinical finding of an extensive lumbosacral MMC, not recognized prenatally, the child was immediately transported to our tertiary care centre. An initial magnetic resonance imaging (MRI) confirmed the finding of a MMC at the level below the fifth lumbar vertebra, where neural tissue herniated into a big dorsal sac. Neither enlargement of the spinal canal nor syringomyelia was found (Fig. 1A). There was elongated neural tissue in the cele, ending in a placode located on the posterior surface. Intracranially, we could see a mild degree of CM2 with reduced volume of posterior cranial fossa and downward herniation of the vermis and reduced cerebrospinal fluid (CSF) space in the foramen magnum. The fourth ventricle was small, but not elongated. No flow artefact was seen in the aqueduct (Fig. 1B). Supratentorial ventricles were small and colpocephalic (Fig. 1C).

On the first day after birth, we performed repair of MMC with primary microsurgical closure of the placode. However, the spinal cord was extremely elongated, and it was not possible to perform a primary suture of the dura, as there was inadequately small space in the spinal canal for the whole volume of the herniated spinal cord. Therefore, we only performed a water-tight suture of lumbosacral fascia, followed by meticulous closure of subcutaneous tissue and skin.

There was no need for cerebrospinal fluid diversion during the postoperative course and further follow-up.

**Fig. 2:**
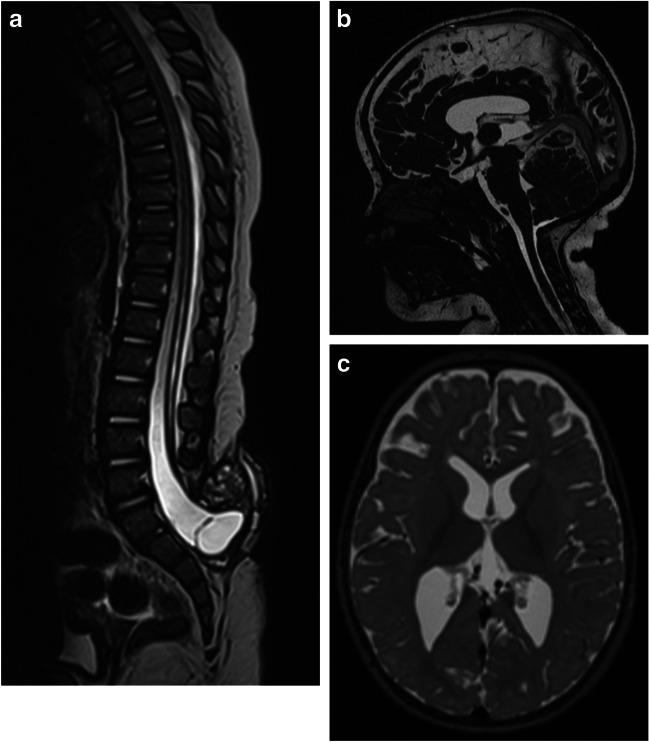
MRI taken 8 months after MMC repair showed **A** tethered spinal cord with tiny syringomyelia from Th11 through L5. **B** Although the tectum still was beaked and the posterior cranial fossa was small, there was no longer any visible herniation of vermis into the foramen magnum, and the position of pons and medulla oblongata was normalized, with adequate CSF space around the medulla oblongata in the foramen (3D CISS). **C** Supratentorial ventricles were slightly asymmetrical and only moderately enlarged (T2WI)

At 8 months of age, the child was active and had some function in the lower extremities, yet significantly reduced. Due to the loss of sphincter control, clean intermittent catheterization was introduced. A follow-up MRI taken at this time showed a tethered cord as expected and minor syringomyelia from Th11 through L5 (Fig. 2A). Surprisingly, although the tectum still was beaked and the posterior cranial fossa was small, other radiological features of CM2 had completely regressed: there was no longer any visible herniation of vermis into the foramen magnum, and position of pons and medulla oblongata was normalized; there was also adequate CSF space around medulla oblongata at the level of the craniocervical junction (Fig. 2B). Supratentorial ventricles were slightly asymmetrical and only moderately enlarged (Fig. 2C).

**Fig. 3:**
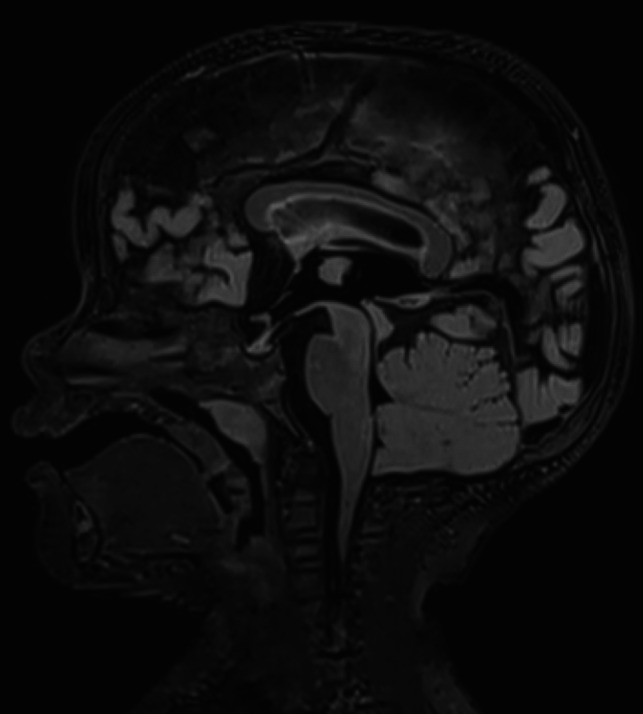
MRI taken 2 years after MMC repair still shows full regression of radiological signs of CM2

The observation of completely regressed radiological signs of CM2 was confirmed by a follow-up MRI taken at the age of 2 years (Fig. 3).

## Discussion

The main finding from the present study is that following postnatal MMC repair, we could observe radiological regression of CM2 in only one single case, representing 6% of children with MMC and associated CM2 in the present series. We also find it interesting in our particular case that the exceptionally large herniation and elongation of the spinal cord were associated with a disproportionally mild degree of CM2 and no significant hydrocephalus. The latter fact is particularly striking, as ventricular shunting could to some extent explain the resolution of CM2 by restoration of hydrodynamic forces inside the posterior fossa.

Unlike Chiari malformation type 1 (CM1), in which several different pathophysiological mechanisms leading to tonsillar ectopy may be identified [18–20], the aetiology and pathophysiology of CM2 are exclusively linked to spinal dysraphism (i.e. the MMC) and appears more complex. The herniation of the spinal cord—typically through a bony defect in the lumbosacral region—and decreased intraspinal pressure cause traction of the rhombencephalon downwards into the enlarged foramen magnum [2]. Even after surgical repair of the MMC, persisting and potentially increasing tethering of the spinal cord—along with concomitant hydrocephalus—may aggravate the crowding of herniated hindbrain structures in the foramen magnum, even requiring surgical decompression in some symptomatic cases.

CM2 is a consistent radiological finding in the majority of children with MMC [2–4], though not all [21], identifiable to some degree on MRI even after successful treatment of MMC and associated hydrocephalus, and despite little clinical significance in the majority of cases [3, 4, 16]. In the present series, surgery for CM2 was necessary in only 19% of children during the observation time. However, CM2 is still the commonest cause of mortality of small children with MMC [5, 6], and close follow-up of these children is therefore necessary, with a focus on hindbrain function and adequate management of spinal cord tethering and hydrocephalus.

Spontaneous regression of hindbrain herniation in CM2 is difficult to observe in real life, as it is associated with meningomyelocele (MMC) that is surgically repaired either pre- or early postnatally. Furthermore, the majority of children require early diversion of CSF.

CM2 was considered to be an irreversible pathoanatomical finding until the first observation of Tulipan et al. [9] that prenatal (i.e. intrauterine) MMC repair may reverse the preexisting hindbrain herniation. Indeed, the proportion of infants without hindbrain herniation was later found to be higher after prenatal (36–39%) than postnatal (4–13%) MMC repair [8, 13, 14]. The reversibility rate following open prenatal MMC repair was reported to be 15–81.5% [11, 13, 14] and even higher with the use of endoscopic technique [11, 12]. Accordingly, fewer children with prenatal MMC repair require surgery for symptomatic CM2 during early childhood [22].

However, regression of CM2 in children undergoing postnatal repair of MMC was specifically reported in only a few previous studies and case reports [15–17], including one larger recent study from 47 children with CM2, where the reversibility rate of CM2 was as much as 40.4% [3] (Table 2). This seems to be an unexpectedly high number, which we could not confirm from our patient cohort, where we observed only one single case out of sixteen newborns with CM2 (6%).

**Table 2:** Studies previously reporting regression of Chiari malformation type 2 in children with postnatally repaired meningomyelocele (excluding single case reports)

Study	*n*	HC requiring treatment	CM2	CM2 requiring treatment	CM2 regression rate	Follow-up
	**MMC**					
Morota et Ihara, 2008 [15]	20	17 (85%)	18 (90%)	3 (17%)	?^1^	Mean 46.5 days
Hashiguchi et al., 2016 [16]	28^2^	19 (68%)	18 (64%)	2 (7.1%)	?	Mean 36.7 months
Beuriat et al., 2017 [3]	61	33 (54%)	47 (77%)	7 (25%)	19 (40%)	Mean 8.1 years
Present study	18	15 (83%)	16 (89%)	3 (19%)	1 (6%)	Mean 59 months

^1^No regression rate was given, only “ascent of the cerebellar tonsils”, observed in 15 cases (83%)

^2^Out of whom three children died or were lost to follow-up after 2, 7 and 18 months, respectively

*CM2*, Chiari malformation type 2; *HC*, hydrocephalus; *MMC*, meningomyelocele

The main limitation of our study is the limited size of the patient sample, reflecting the rarity of new MMC cases in high-income countries, where routines for folate acid supplementation and prenatal screening are well established. In addition, the radiological follow-up in our study is shorter than in the largest study on this topic published so far [3]. However, the significance of follow-up duration for observation of regression of CM2 is uncertain, as in another study the level of cerebellar peg rapidly ascended within the first 6 months, and further gradual ascent was noted over the ensuing 2–3 years [16]. Our only observation of spontaneous regression of CM2 was also made after only 8 months.

## Conclusions

In our experience, spontaneous regression of CM2 in children with postnatally repaired MMC occurs quite rarely. Pathophysiological mechanisms behind the development of CM2 in children with spinal dysraphism remain unclear, but our single observation probably supports the hypothesis of an association between the downward displacement of the hindbrain in CM2 and the low intraspinal pressure secondary to CSF leakage in children born with MMC.

## Data Availability

No datasets were generated or analysed during the current study.
